# H. Reich

**Published:** 1913-03

**Authors:** 


H. Reich, Tübingen (*Zent. fur Chir.*, November 9, 1912), reports the successful transplantation of a section of the ear with its cartilage to fill the defect caused by the excision of a cancer of the left naris.

